# Impact of treatment of molar-incisor hypomineralisation on children’s oral health-related quality of life: a systematic review

**DOI:** 10.1007/s40368-025-01114-6

**Published:** 2025-09-30

**Authors:** R. P. P. Hoogeveen, E. Momayez, C. C. Bonifacio, D. J. Manton, D. Hesse

**Affiliations:** 1 Present Address: Academic Center for Dentistry Amsterdam, Amsterdam, Netherlands; 2 University Medical Center Groningen, Groningen, Netherlands

**Keywords:** Children, Molar-incisor hypomineralisation, Dental treatment, Oral health-related quality of life, Systematic review

## Abstract

**Purpose:**

The present systematic review aimed to investigate the impact of dental treatment for molar-incisor hypomineralisation (MIH) on the oral health-related quality of life (OHRQoL) in children.

**Methods:**

A literature search was conducted in four databases (Medline/PubMed, Embase, Scopus, and Google Scholar). Two independent reviewers selected studies amongst children with MIH who had received dental treatment, with OHRQoL assessed both before and after interventions. Data were critically summarised following the Synthesis Without Meta-analysis guideline. The risk of bias was assessed using the Cochrane ROB-2 tool, and the certainty of evidence was assessed using the GRADE approach.

**Results:**

From the initial 460 identified studies, five were included. These studies included a total of 491 children aged 3–16 years (258 females and 233 males), each with at least one MIH-affected tooth. Qualitative synthesis indicated an improvement in OHRQoL in children following MIH treatment. However, the included studies exhibited potential biases in specific domains, and the certainty of evidence was rated as low.

**Conclusion:**

Treatment of MIH-affected teeth positively impacts the OHRQoL of children.

## Introduction

Molar-incisor hypomineralisation (MIH) is defined as a qualitative developmental defect of dental enamel that affects one or more first permanent molars (FPMs), often affecting permanent incisors (Weerheijm et al. 2001). Affected teeth have clinically visible demarcated hypomineralised lesions, which vary in colour from white/creamy to yellow/brown. Although the enamel thickness remains normal, its structural quality is compromised due to decreased mineral density, and increased concentrations of carbonates and proteins when compared to sound enamel (Elhennawy et al. 2017). The MIH-affected enamel is porous and can break easily, especially once it is exposed to masticatory forces (Weerheijm 2004).

MIH can influence the oral health-related quality of life (OHRQoL) of affected children. In severe cases, symptoms such as hypersensitivity/pain occurs commonly, leading to difficulties in daily activities, such as toothbrushing, eating, and drinking (Gevert et al 2024). Persistent discomfort may have broader implications for the child’s psychosocial development and day-to-day functioning, both at home and in educational settings (Altner et al. 2022). Moreover, when anterior teeth are affected, MIH can cause aesthetic concerns due to changes in surface structure and colour of the hypomineralised lesion, rapid caries lesion development, and enamel breakdown (Gevert et al. 2024). These issues may negatively influence the emotional state of the child (Kisacik et al. 2024; Sekundo et al. 2024).

Several studies have evaluated the OHRQoL of children with MIH, and two recent systematic reviews of observational studies have suggested that the presence of MIH negatively impacts the OHRQoL of affected children (Jawdekar et al. 2022; Amrollahi et al. 2023). Furthermore, some individual longitudinal studies have suggested an improvement in OHRQoL in children with MIH following dental treatment (Bekes et al. 2021; Zhao et al. 2023); however, it is unknown whether different treatment approaches consistently result in improved OHRQoL for these children. Therefore, the present systematic review aimed to investigate the impact of dental treatment for MIH on the OHRQoL in children.

## Materials and methods

### Protocol registration

For the present systematic review, the Preferred Reporting Items for Systematic Reviews and Meta-Analyses (PRISMA) guidelines were followed (Page et al. 2021), and the protocol was registered in PROSPERO (registration number: CRD42024521364).

### Research question and search strategy

The present review was designed to answer the research question: Does dental treatment of MIH-affected teeth result in improved OHRQoL of affected children?

**Fig. 1:**
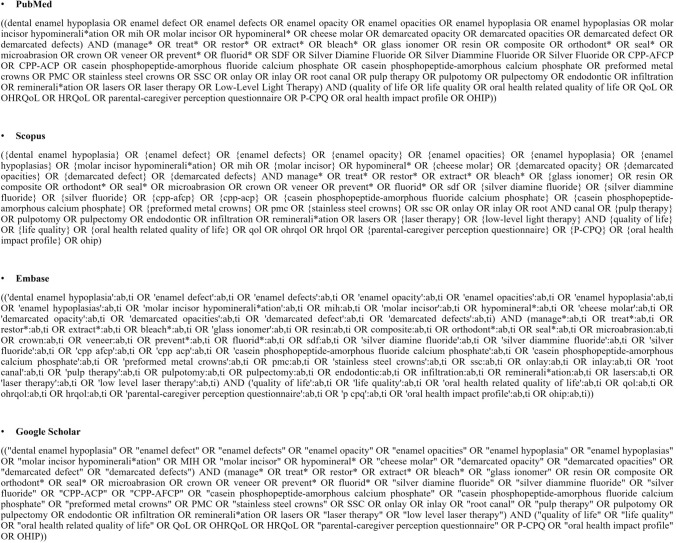
Search strategy used in each database

The literature search was conducted in four databases (Medline/PubMed, Embase, Scopus, and Google Scholar) in February 2024, and the last update was performed in February 2025. The keywords used in each database can be found in Fig. 1. No filters or restrictions (e.g., language, publication type, or publication date) were applied during the database searches to ensure a comprehensive literature retrieval. Authors of published papers were not contacted for additional information. Studies obtained from the search strategy were entered into RAYYAN, an online web application for the screening of literature reviews. Duplicate removal was performed electronically using RAYYAN.

### Study selection and eligibility criteria

First, all titles and abstracts of the retrieved studies were assessed following the inclusion criteria adopted in this review. After that, the full text of the eligible studies was reviewed according to the exclusion criteria. The inclusion and exclusion criteria adopted in the present review can be found in Table 1.

**Table 1:** Inclusion and exclusion criteria adopted in this review

Inclusion criteria	Exclusion criteria
● Studies involving children ● Presence of Molar Incisor Hypomineralisation (MIH) ● Dental treatment performed ● Oral Health-Related Quality of Life (OHRQoL) assessed ● Randomised or non-randomised clinical studies	● Children with systemic diseases ● Children with dental fluorosis, amelogenesis imperfecta, hypoplasia or hypomineralization other than MIH ● Studies with sample overlapping; in this case, the most recent study or that had a more complete description of methodology and results was selected ● Clinical studies with dropout > 30% ● Studies that do not report OHRQoL before and after treatment ● Studies not distinguishing between dental caries and MIH

Independently, two reviewers (EM and RPPH) screened the titles/abstracts of papers according to the inclusion criteria. Subsequently, the same reviewers assessed the full texts of potentially eligible publications. In case of disagreement, discussions with other two independent examiners (DH and CCB) took place, and consensus was achieved. The agreement between the two reviewers was evaluated using 10% of the papers initially selected. The inter-rater reliability was calculated according to the Cohen’s Kappa coefficient (*K* = 0.79)

### Data extraction

The same reviewers (EM and RPPH) also independently extracted data from the included studies using a standardised data collection form. This step was cross-checked by the other two reviewers (DH and CCB). The following data were extracted: publication details (authors, year, and country), study design, sample characteristics (participant number, age, sex), methodology aspects (group formation, clinical intervention), outcomes assessed (instruments used, time of evaluation, follow-ups), and main findings.

### Risk-of-bias assessment and certainty of the evidence

The same reviewers (EM and RPPH) evaluated the risk of bias and certainty of the evidence amongst the included studies using the ROB-2 tool (Sterne et al. 2019), which is structured into six domains: bias arising from the randomisation process, deviations from intended interventions, missing outcome data, measurement of outcomes, selection of the reported results, and overall bias. Each domain was rated as either ‘low risk’, ‘some concerns’, ‘high risk’, or ‘no information’. A study was considered to have a low risk of bias when all domains were rated as low risk. If there were concerns in one or more domains, the study was classified as having ‘some concerns’. When any domain was assessed as having a high risk of bias, the study was considered to have an overall high risk of bias.

Additionally, the certainty of the evidence was evaluated using the Grading of Recommendations Assessment, Development and Evaluation (GRADE) approach. For that, the GRADEpro GDT approach was used to rate the certainty of the evidence for the main outcome (Schünemann et al. 2013). This assessment considered the following criteria: study design, risk of bias, consistency, indirectness, imprecision, and publication bias (Atkins et al. 2004).

## Results

**Fig. 2:**
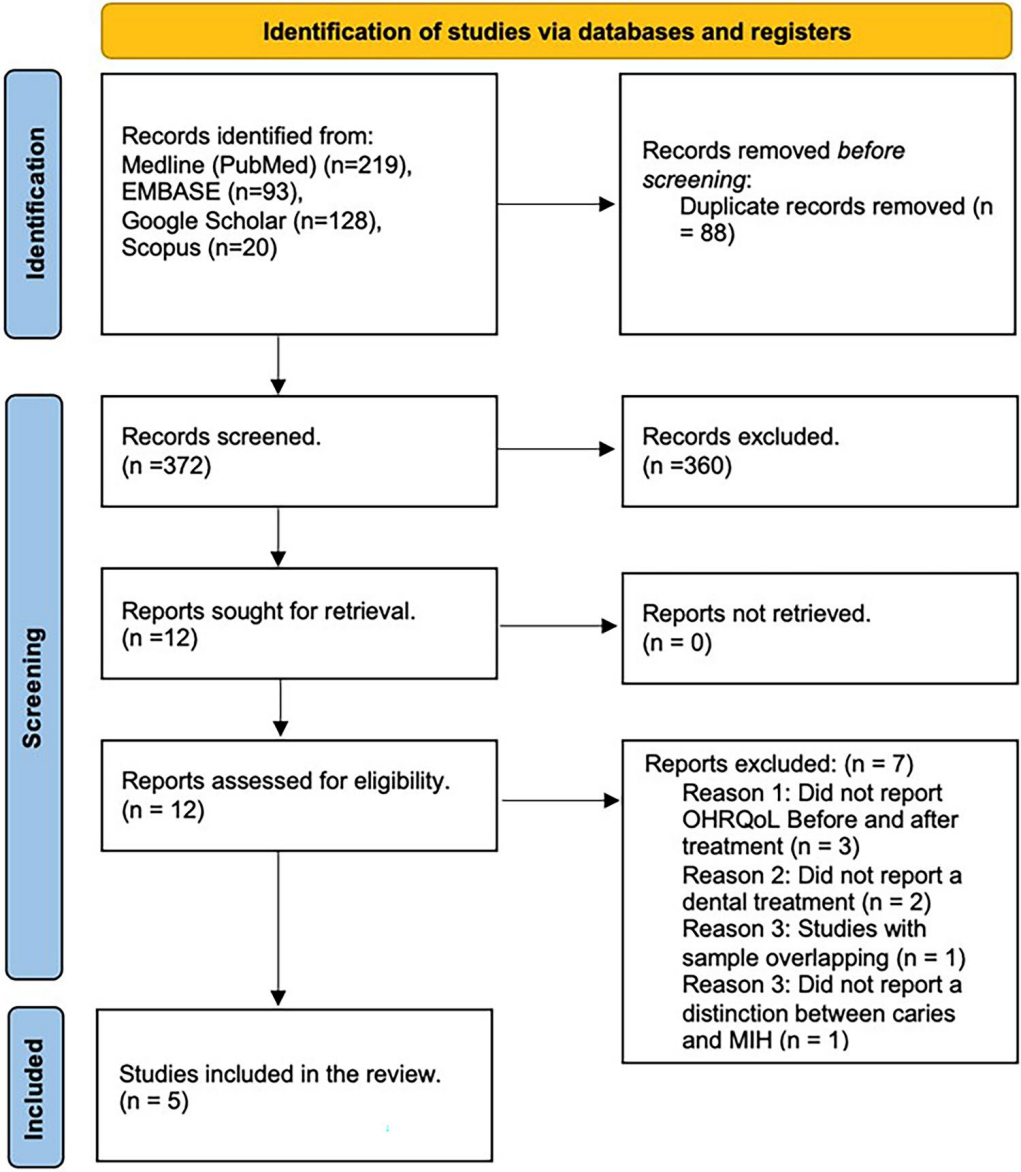
Preferred reporting items for systematic reviews and meta-analyses flowchart showing the selection process

Initially, the electronic search retrieved 460 articles; after removal of duplicates, a total of 372 unique studies were identified. After screening and selection based on title and abstracts, 12 articles were included for full text analysis. Finally, seven articles were excluded, resulting in five articles included in the present review. A study selection flow diagram including the reasons for exclusion of full texts is illustrated in Fig. 2.

### Characteristics of included studies

The characteristics of the included papers are depicted in Table 2. The included studies were conducted in five different countries (Germany, Austria, UK, China, and Turkey) and were published between 2018 and 2023. Collectively, the studies investigated a total of 491 children presenting with at least one MIH-affected tooth. The age of participants ranged from 6 to 16 years, comprising 258 females and 233 males.

**Table 2.** Characteristics and summary of results of the included papers

Author, year, country, type of study	Sample (♀, ♂)	Mean age ± SD years (range)	Method of assessment treatment need (diagnose/severity)	Questionnaires used	Method of questionnaire application	Time of application	Type of teeth/type of treatment	Total scores of OHRQoL of children before and after treatment (mean ± SD)	Main results
Hasmun et al. 2018, UK, Non-randomised clinical study	55/38	10.95 ± 2.54 (7–16)	Criteria proposed by Weerheijm 2003/NR	C-OHIP-SF19	By participant, checked by clinicians for completeness	Before and 1 month after treatment	Anterior teeth/ Microabrasion (Opalustre, Ultrandent), Resin infiltration (ICON, DMG), Teeth whitening (Opalescence, Ultradent), Resin composite restorations (Filtek, 3M)	Overall C-OHIP score before treatment 47 ± 9.29. After treatment 58.24 ± 9.42.*	Improvement of self-reported OHRQoL following minimally invasive aesthetic treatment.
Bekes et al. 2021, Austria and Germany, Non-randomised clinical study	18/20	7.5 ± 1.6 (6–10)	EAPD criteria (Weerheijm et al 2003)/MIH-TNI 3 AND SCASS: 2 or 3	CPQ 8-10 (German version)	By participant	Before and 1, 4, 8, and 12 weeks after treatment	Posterior teeth/ Resin-based sealant (Scotchbond Universal + Clinpro, 3M), Glass ionomer cement sealant (Ketac Universal, 3M)	CPQ before treatment 14.7 (± 5.9), one week after treatment 6.4 ± 4.7, 12 weeks after treatment 2.7 ± 3.2.	Sealing of hypersensitive MIH-affected molars revealed a significant improvement of OHRQoL immediately and throughout the 12-week follow-up.
Altner et al. 2022, Germany, Case-control study	103/107	9 ± 2 (7–11)	EAPD criteria (Weerheijm et al 2003)/Mathu-Muju and Wright 2006	CPQ-G8–10 (German version)	By participant	Before and after treatment	Anterior and posterior teeth/ Different therapies: No therapy, non-invasive, minimal invasive, invasive, endodontic, prosthodontic, extraction and combinations	Total CPQ (MIH) before treatment 17.88 ±10.6, after treatment: 7.54 ± 4.75; Total CPQ (caries) before treatment: 13.88 ±14.23, after treatment: 7.3 ± 6.71)	Greater relative improvement of OHRQoL in patients with MIH compared to patients with severe dental caries.
Tugcu et al. 2022, Turkey, Non-randomised clinical study	24/16	11.85 ± 1.02 (11–14)	EAPD criteria (Weerheijm et al 2003)/B-code MIH-TNI 2a/b/c	CPQ11–14 (Turkish version)	By participant	Before and 6 months after treatment	Posterior teeth/ Glass hybrid cement restorations (Equia Forte, GC Corp.)	Overall CPQ score before treatment 33.27 ± 16.46 and after treatment 11.67 ± 11.21	Treating MIH-affected molars with Glass Hybrid restoration after SCR improved these children’s OHRQoL (no gender differences).
Zhao et al. 2023, China, Randomised controlled trial	58/52	10.30 ± 1.73 (11–14)	EAPD/VAS	OHIP-14 (Chinese version)	By participant	Before and 5 months after treatment	Anterior and posterior teeth/ Control group, Desensitizer (Gluma, Kulzer), Er:YAG laser therapy, combination of Er:YAG laser therapy + Desensitizer (Gluma, Kulzer)	Overall OHIP before treatment: 17.61 ± 4.22, after treatment: 3.54 ± 2.67.**	All treatment modalities showed an improvement in the OHRQoL of children. Combination therapy of Er:YAG laser and GLUMA desensitizer had greater desensitising effects and OHRQoL improvement.

*SD* Standard deviation, *OHRQoL* Oral health-related quality of life, *EAPD* European Academy of Paediatric Dentistry, *MIH-TNI* Molar Incisor Hypomineralisation-Treatment Need Index, *SCASS* Schiff Cold Air Sensitivity Scale, *VAS* Visual Analogue Scale, *NR* not reported: *SCR* Selective Caries Removal

^*^in the study of Hasmun et al. (2018), a higher C-OHIP score indicates a better OHRQoL

^**^In the investigation of Zhao et al. (2023), scores were reported separately for the different treatment groups. The mean ± SD reported in the table refers to the group where Er:YAG laser and GLUMA desensitizer was applied

Regarding MIH diagnosis, one study (Hasmun et al. 2018) used the criteria proposed by Weerheijm (2003), three studies (Bekes et al. 2021; Altner et al. 2022; Tugcu et al. 2022) applied the EAPD criteria (Weerheijm et al. 2003), and one study (Zhao et al. 2023) mentioned the use of EAPD criteria without further specification. A variety of treatment modalities were applied across the studies. These included restorative treatment with glass hybrid restorations following selective carious tissue removal (Tugcu et al. 2022), application of resin-based sealants in combination with adhesive system (Bekes et al. 2021), and a range of minimally invasive techniques, including microabrasion, resin infiltration, tooth whitening, and resin composite restorations (Hasmun et al. 2018). Additionally, laser therapy employing erbium-doped yttrium aluminium garnet (Er:YAG laser), in combination with desensitising agents, was reported (Zhao et al. 2023). Other interventions, such as restorative procedures, extractions, endodontic treatment, and prosthodontic approaches, were also described (Altner et al. 2022).

The instruments used to evaluate the OHRQoL of children were the Child Perceptions Questionnaire (CPQ), Oral Health Impact Profile (OHIP) and the Child Oral Health Impact Profile (C-OHIP). The questionnaires used were validated in the language in which they were applied, and the most commonly used application method was asking the participants to answer the questions themselves.

### Individual results of the studies

Total OHRQoL scores were measured both before and after treatment in all included studies. Three studies (Altner et al. 2022; Tugcu et al. 2022; Bekes et al. 2021) also evaluated specific OHRQoL domains separately—namely, oral symptoms, functional limitations, emotional well-being, and social well-being. One study (Hasmun et al. 2018) used C-OHIP, which includes domains of oral health, functional well-being, and socio-emotional well-being. Another study (Zhao et al. 2023) used the OHIP-14, covering the domains psychological disability, social disability, handicap, psychological discomfort, and physical pain.

All questionnaires were administered both pre- and post-treatment, with post-treatment assessment intervals ranging from 1 week to 6 months. The main findings indicated an overall improvement in CPQ/(C-)OHIP scores, suggesting a positive impact on OHRQoL following MIH treatment (Table 2). A more detailed insight emerged from the domain-specific analysis, with all domains showing improvement post-treatment. Notably, the most substantial improvement was observed in the emotional well-being domain (Table 3).

**Table 3.** Mean (SD) of CPQ/ (C-)OHIP scores per domain before and after treatment

Author		Oral symptoms mean ± SD	Functional limitations mean ± SD	Emotional well-being mean ± SD	Social well-being mean ± SD	Psychological disability mean ± SD	Social disability mean ± SD	Handicap mean ± SD	Psychological discomfort mean ± SD			Physical pain mean ± SD
Hasmun et al. 2018	*Before ***After	11.26 ± 2.78 14.15 ± 3.34	13.28 ± 2.59 14.16 ± 1.87	22.46 ± 7.67 29.92 ± 6.42	22.46 ± 7.67 29.92 ± 6.42	N.A.	N.A.	N.A.		N.A.	N.A.	
Bekes et al. 2021	Before After	7.5 ± 3.0 1.6 ± 1.9	4.5 ± 1.6 0.6 ± 1.1	1.8 ± 1.9 0.2 ± 0.6	0.9 ± 1.9 0.3 ± 0.6	N.A.	N.A.	N.A.		N.A.	N.A.	
Altner et al. 2022	Before After	6.36 ± 2.99 3.16 ± 1.7	4.9 ± 3.33 2.08 ± 1.9	5.06 ± 4.01 1.97 ± 2.19	1.55 ± 2.06 0.33 ± 0.79	N.A.	N.A.	N.A.		N.A.	N.A.	
Tugcu et al. 2022	Before After	9.2 ± 4.24 3.52 ± 2.82	7.2 ± 5.03 3.35 ± 2.79	11.87 ± 8.73 3.18 ± 5.87	5.0 ± 6.0 1.62 ± 4.05	N.A.	N.A.	N.A.		N.A.	N.A.	
Zhao et al. 2023	**Before After	4.04 ± 1.99 0.93 ± 0.94	N.A.	N.A.	N.A.	1.96 ±1.1 54 ± 0.64	1.93 ± 1.12 0.32 ± 0.61	1.25 ±1.21 0.61 ± 0.83		3.43 ±1.81 0.61 ±0.88	4.04 ±1.99 0.93 ±.94	

*SD* Standard deviation, *N.A.* Not Applicable

^*^C-OHIP-19 comparative category. Emotional well-being and social well-being is 1 category

^**^OHIP-14 comparative category

^***^ In the study of Hasmun et al. (2018), a higher C-OHIP score indicates a better OHRQoL

### Risk of individual bias assessment

**Fig. 3:**
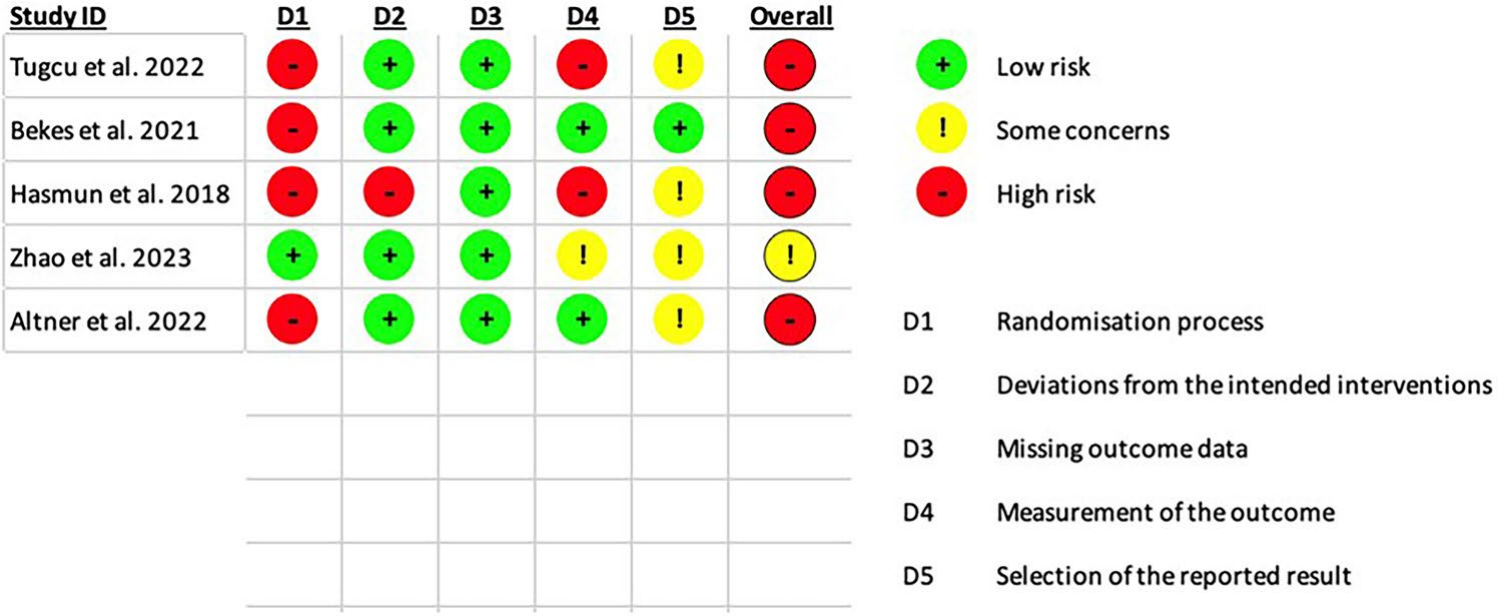
Risk of bias of the included studies

After methodological quality assessment, four studies (Tugcu et al. 2022; Altner et al. 2022; Bekes et al. 2021; Hasmun et al. 2018) were considered as presenting a high risk of bias, due to a low score for the “randomisation process”. One study (Zhao et al. 2023) showed some concerns by scoring low for the domains “measurements of the outcome” and “selection of the reported results”. The detailed assessment of bias per domain and overall risk of bias is illustrated in figure 3.

### Certainty of evidence

The detailed GRADE evaluation is summarised in Table 4. The overall certainty of the evidence regarding the impact of dental treatment for MIH on children’s OHRQoL was rated as low, mainly due to the limited number of available studies, methodological limitations, and imprecision. When outcomes were considered separately, the certainty of evidence was judged to be low for improvements in pain/discomfort, very low for improvements in functional limitations, and low for aesthetic and psychosocial outcomes.

**Table 4:** GRADE summary of evidence for the impact of dental treatments for MIH on OHRQoL

Outcome	No. of studies (design)	Risk of Bias	Inconsistency	Indirectness	Imprecision	Publication bias	Certainty of evidence
Overall OHRQoL improvement after MIH treatment	5 (1 RCT, 4 observational)	Serious (due to concerns in non-RCTs)	Not Serious	Not serious	Serious (small sample sizes in most studies)	Undetected	⊕⊕◯◯ Low
Pain/discomfort reduction	2 (1 RCT, 1 observational)	Some concerns	Not Serious	Not serious	Not serious	Undetected	⊕⊕◯◯ Low
Functional improvement (chewing, hypersensitivity)	2 observational	Serious	Not Serious	Some concerns (proxy reporting)	Serious	Undetected	⊕◯◯◯ Very low
Aesthetic/psychosocial improvement	3 observational	Serious	Not Serious	Not serious	Serious	Undetected	⊕⊕◯◯ Low

*RCT* Randomised clinical trials

## Discussion

The present systematic review aimed to investigate the impact of dental treatment of MIH on the OHRQoL of children. The included studies demonstrated an overall improvement in OHRQoL following treatment. The most notable changes were observed in the domains ‘oral symptoms’ and ‘socio-emotional wellbeing’; which can be explained by the nature of the treatments performed. Treatment modalities applied in posterior teeth focussed mainly on pain and hypersensitivity management, and food intake; and clinical improvements were reflected in higher scores in the ‘oral symptoms’ domain (Altner et al. 2022; Tugcu et al. 2022; Bekes et al. 2021; Zhao et al. 2023). On the other hand, treatments involving anterior teeth were aimed mainly at improving aesthetics, which led to increased self-confidence amongst the children, reflected in a positive impact on the ‘socio-emotional well-being’ domain (Hasmun et al. 2018). These observations align with the findings of Shields et al. (2025), who reported that children with MIH-affected incisors exhibited poorer OHRQoL compared to both unaffected peers and those with MIH limited to molars. Although their results were associated with wide confidence intervals and should therefore be interpreted with caution, they nonetheless underscore the significant psychosocial impact of incisor involvement. This distinction highlights the importance of considering the specific location of MIH lesions in both future research and clinical evaluations.

The included studies did not assess the emotional status of the participants before treatment. This is an important aspect, as pain and anxiety may negatively impact mental well-being, which in turn could result in a more adverse perception of OHRQoL (Thompson et al. 2016; Ladewig et al. 2018). This may have influenced the participants’ responses to the questionnaires, thereby affecting the reliability of the data and the overall findings. Furthermore, there is some evidence, suggesting that children with MIH are more likely to exhibit behaviour management problems during dental treatment compared to their unaffected peers, which is most probably related to the increased caries experience, higher frequency of restorative procedures, and difficulty with effective local anaesthesia experienced by these individuals (Reis et al. 2023; Shields et al. 2024). Although this evidence is of very low quality, it highlights the importance of implementing an individualised and effective treatment strategy for the management of MIH. By reducing the need for re-interventions, the functional and aesthetic needs of children with MIH can be addressed, and also associated behavioural issues potentially prevented.

Another important aspect is the relatively short duration of follow-up across the included studies, which ranged from immediately post-treatment to 6 months thereafter. Longer term follow-up is essential to provide a more comprehensive evaluation of treatment effectiveness, assess potential long-term outcomes and complications, and improve the reliability and generalisability of the findings to broader populations (Fitzpatrick et al. 2018). Additionally, the cost-effectiveness of the proposed treatments could be evaluated in the future, as this is an essential factor to enhance allocation of financial resources. Incorporating economic evaluations could support strategies aimed at improving oral health outcomes in individuals affected by MIH, whilst also helping to prevent overtreatment and minimise negative consequences for healthcare and insurance systems (Beck et al. 2022).

The different index systems used to diagnose or report the severity of MIH in the included studies may have directly influenced the overall OHRQoL scores. Due to this variation, it is likely that more severe cases of MIH result in greater discomfort and, consequently, higher CPQ scores, especially before treatment (Bekes et al. 2021). Indeed, a recent cross-sectional study investigating the impact of MIH on the OHRQoL of Australian schoolchildren showed that participants with severe MIH experienced a significantly greater average negative impact on OHRQoL compared to those without MIH (Shields et al. 2025). Moreover, many individuals with MIH also present with coexisting oral conditions such as carious lesions, which may confound the interpretation of results and obscure the specific impact of MIH. Therefore, there is a clear need for standardisation in both diagnostic criteria and severity classifications of MIH, and future research should aim to assess treatment outcomes separately for mild and severe cases (Shields et al. 2024). In addition, MIH phenotypic classifications—such as incisor involvement—should be considered, as recent evidence suggests that severe MIH, particularly when incisors are affected, may be associated with poorer OHRQoL (Shields et al. 2025). Furthermore, although improvements in children’s OHRQoL following MIH treatment were observed in the present study, caution must be exercised when interpreting and generalising these findings. This is particularly important, because the CPQ, used in most of the included studies, has been validated for conditions such as dental caries, cleft lip and/or palate, and orthodontic issues, but not specifically for MIH.

The majority of the studies included in the present review were assessed as having a high risk of bias, primarily due to limitations in study design and the absence of proper randomisation procedures. However, true randomisation is inherently unfeasible in MIH research, as the condition itself cannot be assigned randomly. Moreover, treatment strategies were often individualised based on the clinical needs of each individual, making it difficult to include comparison or control groups within these studies. To mitigate bias in future research, it is important to recruit participants with comparable levels of MIH severity and to account for potential confounding variables, such as pre-treatment pain or hypersensitivity, anxiety, age, caries risk, and socioeconomic status (Tripepi et al. 2010). Additionally, efforts should be made to minimise variability stemming from self-reported outcomes by incorporating objective evaluation tools, such as the SCASS and MIH-TNI indices, which can help standardise outcome assessment and reduce bias (Tripepi et al. 2010). Finally, to further strengthen the validity of findings, the involvement of independent and, where feasible, blinded operators, examiners, and interviewers is recommended, as this can enhance the accuracy of estimating both predictive and causal effects.

Heterogeneity in oral health assessment instruments amongst the selected studies significantly constrained the present systematic review, limiting the ability to conduct meta-analysis on the impact of MIH treatment on OHRQoL. Variations in sample sizes, participant ages, intervention types, evaluation times, and follow-up periods prevented meaningful quantitative comparisons of OHRQoL improvements before and after treatment. The clinical and statistical diversity across studies made it difficult to draw definitive conclusions about how different treatments affect various dimensions of children’s OHRQoL. Although no language restrictions were applied in our search strategy, all included studies were published in English. This reflects the availability of studies in this field rather than a deliberate exclusion of non-English publications; nonetheless, it may represent a potential source of language-related bias. The certainty of the available evidence, using the GRADE approach, might be considered as a limitation, since the certainty was rated overall as low across the studied outcomes. This reflects the small number of available studies, the predominance of non-randomised designs, and concerns about methodological quality and imprecision. Only one randomised clinical trial contributed moderate-certainty evidence for short-term improvements in OHRQoL (Zhao et al. 2023). Consequently, whilst the available studies consistently suggest that dental treatment for MIH is associated with improvements in children’s OHRQoL, the overall strength of the evidence remains limited.

Despite these challenges, several strengths can be highlighted in the present review. To the best of our knowledge, this is the first study to systematically summarise and assess the existing evidence on the OHRQoL in children with MIH before and after treatment. This approach not only provides valuable insights into the impact of treatment from the patient’s perspective but also supports clinical decision-making and disseminates knowledge to professionals, MIH-affected children, and their families. The review’s methodological rigour, particularly in data analysis and risk-of-bias assessment, is a key strength. The use of the RoB-2 tool (Sterne et al. 2019), endorsed by the Cochrane Collaboration, helped ensure a rigorous evaluation of bias in the included studies. Additionally, the authors employed a comprehensive search strategy, encompassing multiple databases with no restrictions on publication date, which enhances the relevance of the findings. As such, the present review can increase practitioners’ awareness of MIH and the importance of treating affected teeth to improve children’s OHRQoL.

## Conclusions

Based on the studies included in this systematic review, treatment of MIH-affected teeth had a positive impact on the OHRQoL of children. However, given the limitations and the high risk of bias in most of the included studies, conclusions should be drawn with caution.

## Data Availability

No datasets were generated or analysed during the current study.
